# Transcriptomic Analysis of Peripheral Blood Mononuclear Cells During *Ostertagia ostertagi* Infection in Cattle Highlights a Generalized Host Immune Reaction

**DOI:** 10.3390/biology14081034

**Published:** 2025-08-12

**Authors:** Damarius S. Fleming, Mariam Bakshi, Peter Thompson, Ethiopia Beshah, Wenbin Tuo

**Affiliations:** Animal Parasitic Disease Laboratory, Beltsville Agricultural Research Center, USDA-ARS, Beltsville, MD 20705, USA; mariamb0517@gmail.com (M.B.); peter.thompson@usda.gov (P.T.); ethiopia.beshah@usda.gov (E.B.); wenbin.tuo@usda.gov (W.T.)

**Keywords:** *Ostertagia ostertagi*, abomasum, macrophages, immune response, cattle, gene expression, PBMC

## Abstract

One of the most concerning ruminant infections is the parasite *Ostertagia ostertagi*. Known commonly as the brown stomach worm, it is ingested by grazing cattle where it then progresses its life stages, occupying the host abomasum and then the intestine, causing illness. This results in lower commercial production and at worst, death of young calves. Over time, anthelmintic treatment has become less efficacious against cattle nematodes. As a result, alternative control strategies are needed. Our study looked to elucidate mechanisms underlying attenuation of the host immune response by examining global immune expression in cattle during infection. To this end, four steers were infected with the third stage larvae (L3) of *O. ostertagi*, then peripheral blood mononuclear cells (PBMCs) were collected weekly for 26 days post-infection (dpi). After sequencing, gene expression was compared between each timepoint. The analyses indicated that the immune responses to *Ostertagia* are targeted to the parasite’s life stages and mimics anti-viral gene expression. Overall, the results showed that *O. ostertagi* led to host immune responses characterized by multiple gene ontology and pathway terms indicating that by 26 dpi the host immune system transitions from fighting the parasite to repairing the host intestine.

## Simple Summary

Parasites of cattle and small ruminants cause health and production problems for stakeholders. Part of the problem is the lessening efficacy of drugs slated for use against gastrointestinal (GI) parasites in ruminants. Alternative control measures are needed such as vaccines. To address this issue, our study examined the immune response in cattle infected by the parasite, *Ostertagia ostertagi*. The goal was to take a closer look at how the infected cattle immune system reacts across different life stages of the parasite. The results of the study showed that the cattle immune response was different depending on the life stage of the parasite. The results also showed that blood drawn from the cattle can be used to understand the effect the parasite is having on the animal stomachs. This difference in immune response can be exploited to create new diagnostic testing for infections and stage of infections. Also, the information on the immune response can be used to develop new treatments that may eliminate the parasite or keep it from progressing from one life stage to another. The results from this study will benefit researchers and veterinarians in crafting new diagnostics and treatments which will in the end benefit cattle and small ruminant stakeholders.

## 1. Introduction

Nematode infections in cattle can be highly deleterious to the production goals of cattle producers. One of the most concerning is the parasitic nematode *Ostertagia ostertagi*. Known commonly as the brown stomach worm, it occupies the cattle abomasum causing illness resulting in reduced productivity, growth, and health [1,2]. Severe cases of *O. ostertagi* can be tolerated in adult cattle but can kill young calves. The parasite’s life cycle contains multiple larval stages (L1–L5) prior to acquiring its adult stage. Transmission occurs when cattle encounter fields harboring *Ostertagia* larvae. During grazing the parasite is ingested in its infective L3 larval stage and then completes its life cycle and reproduction within the cattle abomasum [1,3,4]. Previous studies of early infection by *O. ostertagi* highlighted the use of peripheral blood mononuclear cells (PMBCs) to give insight into the parasite’s ability to elicit local and systemic immune responses [5,6,7]. Throughout the course of *O. ostertagi* and other helminth infections, the host innate immune system, including macrophages, performs antigen-presenting and phagocytic actions and initiates acquired immune responses. Although these actions have been explored in the early stages of *O. ostertagi* infection that occur at the L3 and L4 portion of the parasite life cycle [2,5,8], there is less information on host–parasite interactions during the L5 and adult stages. The ability to gather this information would be crucial in determining the extent of host immune responses during late-stage infections.

The latter stages of *O. ostertagi* infections can limit the nutritional needs of cattle through the appropriation of macro- and micronutrients from the host while modulating cattle immune responses. Studies have shown that different life stages of *Ostertagia* have different expression profiles with some genes even being life stage specific [3]. Previous studies on Fasciola hepatica, a trematode of the lives of cattle and sheep [9,10] have explored gene expression changes during *F. hepatica* GI parasite infections using PBMCs to survey the activation and polarization of M2 macrophages. Mechanisms by which macrophages are used to attack GI parasites, like *F. hepatica* and *O. ostertagi*, locally within tissues have been described [9,10,11], but little has been shown on the circulating macrophages during infection. Although drug treatments exist, time has shown them to become less efficacious [12,13,14]. As a result, alternative control strategies are needed [15], however more studies on the host–parasite interaction are necessary to power alternative methods. Our study looks to elucidate mechanisms underlying overall host immune responses by examining global immune expression during infection. This information will help expound upon, and further, efforts to recognize alternative treatments based on host–parasite interactions at the genomic level in cattle.

## 2. Materials and Methods

### 2.1. Animals and Experimental Design

Holstein steer males (N = 4) with no pasture exposure were subjected to pre-infection bleeding for 2 weeks prior to inoculation with L3 larvae (200,000 L3) of *O. ostertagi*. During the study the animals were housed together and fed a normal diet of hay and grain. The animals were then bled every 7 days post inoculation (dpi) to capture the effect of the parasites on immune gene expression within the host. Each week (7 days) PBMCs were extracted from whole blood obtained from the jugular vein of the 6–7-month-old calves and prepared for RNA sequencing. Parasites used for infection were propagated in-house and were not examined as part of this study. All animal experiment protocols were performed under approval of the Animal Care and Use Committee of Beltsville Agricultural Research Center (BARC) in accordance with relevant guidelines and regulations.

A pure population of *Ostertagia ostertagi* has been maintained in passage through cattle at USDA for years. Every six months to one year, two calves are infected with 200,000 L3. After day 15, buckets of manure are collected daily, checked for the presence of eggs via fecal float, and subjected to culturing by mixing with vermiculite and PBS and storing at ambient temperatures for two weeks while the eggs hatch and mature to L3. The L3s are then collected via large Baermann apparatus. Collected worms are stored at 15 °C until needed. Viability is check monthly by visual examination of a sample of worms. Purity of the culture was last checked in 2023 using the Nemabiome procedure explained in Avramenko et al., 2015 [16].

### 2.2. Sample Collection and Preparation

Blood was drawn from each calf at each proscribed timepoints within the study and prepared for RNA-Seq analysis. Whole blood was collected and processed to separate buffy coat component that held immune system cells. This fraction of the blood was isolated using centrifugation through a density gradient. The RNA was isolated following the Life Technologies protocol. Briefly, 2 × 10^7^ PBMCs were lysed using 1 mL of TRI Reagent (Sigma-Aldrich, Co., St. Louis, MO, USA) and stored at −80 °C freezer until used. The frozen lysates were thawed on ice and 0.2 mL chloroform (Sigma Aldrich, Co., St. Lous, MO, USA) was added followed by vigorous shaking by hand for 15 s, incubation for 3 min at room temperature and centrifugation at 12,000× *g* for 15 min at 4 °C. The upper aqueous phase was transferred into a new 1.5 mL tube, 0.5 mL 2-propanol (Sigma Aldrich, Co., St. Lous, MO, USA) was added, mixed by inversion, incubated at room temperature for 10 min, and centrifuged at 12,000× *g* for 10 min at 4 °C. Then the supernatant was discarded, and the RNA pellet was washed using 1 mL 75% 200 proof alcohol (The Warner-Graham Company, Cockeysville, MD, USA) in DEPC-treated water. Then RNA pellet was air-dried for 5 min and dissolved in RNase-free DEPC-treated water (Quality Biological, Gaithersburg, MD USA). The RNA concentration and quality was determined using RNA 6000 Nano Kit and Agilent 2100 Bioanalyzer system (Agilent Technologies, Waldbronn, Germany) following the manufacturer’s protocol.

### 2.3. Sequencing and RNA Expression Analysis

Sequencing libraries were prepared using an Illumina stranded mRNA prep kit according to manufacturer’s instructions (Illumina, San Diego, CA, USA). Briefly, mRNA was isolated from 500 to 700 ng of total RNA using poly-dT beads. The mRNA was then fragmented, converted to cDNA using reverse transcriptase, attached to Illumina adapter sequences via ligation, and finally given indexes for sample identification and sequence generation using PCR. Libraries were quantified using Qubit HS dsDNA kits and diluted to equal concentrations. Libraries were pooled into a single sequencing reaction and sequenced on an Illumina NextSeq 2000 instrument (Illumina, San Diego, CA, USA) generating 75 bp single-end reads. A total of twenty 75 bp single-end reads for the five total timepoints being compared in the study were generated. Day 0 (T1) served as the control/null sample group compared against 7 dpi (T2), 14 dpi (T3), 21 dpi (T4), and 26 dpi (T5). Sequencing of the PMBCs was carried out in-house using the Illumina NextSEQ. Quality control of the 20 single-end reads was completed using the software FastQC (Galaxy Version 0.74+galaxy0) on the Galaxy.org web platform [17,18]. Quality of the reads was set to a score of 25 or greater to be retained. Reads were mapped to the current *Bos taurus* genome ARS1.3 [19] and GTF file using HISAT2 [20] with default scoring and alignment options. The BAM files were then processed using the program FeatureCounts (Galaxy Version 2.0.8+galaxy0) [21] to obtain the raw gene counts. Using default scoring and alignment options gene expression calculations and statistics were completed using DESeq2 (Galaxy Version 2.11.40.7+galaxy2) [22]. Parameters for DESeq2 were set to default log2fcs for the estimateSizeFactors argument; fit type was set to parametric. Additionally, the options for outlier and independent filtering were set to “yes”. Final gene lists were subjected to an FDR of ≤0.01. Transcriptomic results produced large gene lists and were subjected to a second filter for fold change > than or equal to 1(log2fc) to facilitate gene ontology and pathway analysis. No data was reported for 7 dpi (T2) since it did not meet the FDR thresholds established for the differential expression analysis.

### 2.4. Gene Ontology and Pathway Analysis

The gene expression lists from each comparison were subjected to further exploration using the software gProfiler (2019 update) [23]. The program options were set to use all known genes annotated to *Bos Taurus* to allow for the highest number of query genes to be recognized. An adjusted *p*-value (FDR) of 0.01 was used for significance. Further information regarding pathways and gene effects examined using Ensembl (ver. 114) and NCBI [19,24].

## 3. Results

### 3.1. Gene Expression Analysis Results for 0 Dpi vs. 14 Dpi Timepoint Comparison

Analysis of the gene expression results for T1 (0 dpi) vs. T3 (14 dpi) was compared with the life cycle of *O. ostertagi* at 14 dpi which corresponded with the L4 to L5 larval stage. The stage takes place in the cattle gastric glands or the abomasal lumen where it generated a total of 567 differentially expressed genes. The percentage of upregulated genes equaled 62.8%, almost two thirds of the total. Some of the highest expressed genes included upregulated antiviral and blood hemostasis related genes such as hemoglobin subunit alpha (*HBA*), ubiquitin-like protein ISG15 (*ISG15*), radical S-adenosyl methionine domain-containing protein 2 (*RSAD*), oncostatin-M (*OSM*), and ubiquitin conjugating enzyme E2 L6 (*UBE2L6*). Further inspection of the results uncovered gene expression overlaps with previous studies of cattle experiencing *O. ostertagi* and other gastrointestinal nematode (GIN) infections [5,10,25,26]. An important downregulated immune gene expressed at this timepoint was interleukin 5 receptor subunit alpha (*IL5RA*) (log2fc = −2.32), a receptor for IL-5 known to be a potent Th2 cytokine [19,24]. Genes found to be involved in gastric cell homeostasis [25] were also expressed in the host response, such as the gene heparin binding EGF like growth factor (*HB-EGF*), found to be upregulated at 14 dpi. This gene has been shown to increase in expression during both single and trickle infections of *O. ostertagi* as early as 6 dpi and as late as 60 dpi [25].

The gene *HB-EGF* (log2fc = 1.35) also showed up in several statistically significant pathways that included epithelium cell migration (GO:0010631) and epithelium migration (GO:0090132) (Table 1). which may mark cell turnover or galectin and mucin cell interaction [25]. The *HB-EGF* gene plays a critical role in tissue repair and regeneration and parietal cell dysfunction during *Ostertagia* infections [27]. Another gene family that showed increased expression during infection was the disintegrin and metalloproteinase (ADAM) group [28]. The genes in this group cleave pro-*HB-EGF* to release *HB-EGF* [28]. Genes within this family of metallopeptidases have also been shown to respond to *Ostertagia* infections in host gastric cells [29]. The gene *HB-EGF* also functions as a damage-associated molecular pattern signal (DAMP) and has been shown to effect *O. ostertagi* signaling network components involved in gastric cell homeostasis [27,29]. Other upregulated genes of interest included the genes *JUND* (log2fc = 1.14) and *JUNB* (log2fc = 1.32), which displayed the most gene interactions based on gene ontology (GO) results. Other gene ontology terms showing possible host interactions with the parasite included fibroblast proliferation (GO:0048144) which is related to the host’s ability to repair internal damage [19,24]. Four of the top G.O. terms at 14 dpi included epithelial cell proliferation (GO:0050673), epithelial cell migration (GO:0010631), efferocytosis (KEGG:04148), and apoptotic signaling pathway (GO:0097190). The genes vasohibin 1(*VASH1*) (log2fc = 1.01) and vasohibin 2 (*VASH2*) (log2fc = −1.88), are both part of the 14-dpi epithelial cell proliferation with *VASH1* being a negative regulator of this proliferation while *VASH2*, a positive regulator, is strongly downregulated in comparison.

### 3.2. Gene Expression Analysis Results for 0 Dpi vs. 21 Dpi Timepoint Comparison

The largest change in expression occurred at 21 dpi. At this point the parasite has transitioned from its L5 stage to an adult, causing increased GI damage to the host. The parasitic stage corresponded with a total of 930 differentially expressed host genes with 59% being upregulated. Some of the highest expressed genes included many involved in host response to viral infections, inflammation, and lymphocyte reactions. The increase in DEGs may be related to having more lymphocyte interaction with the inclusion of negative regulation of lymphocyte activation (GO:0051250). The process was upregulated and did not reach significance at 14 dpi. The process seemed to be driven by the gene arginase 1 (*ARG1*) (log2fc = 2.42), which in humans functions as part of the adaptive immune response negatively regulating T-cell proliferation, T-helper 2 production (Th2), and type 2 interferon signaling pathways [19,24]. The results at 21 dpi showed statistical significance in some biological processes not active at 14 dpi such as antioxidant activity (GO:0016209) in which the hemoglobin genes *HBA* (log2fc = 7.61) and *HBB* (log2fc = 6.35) were highly upregulated. The hemoglobin gene expression was likely the result of some red blood cells making their way into the PBMC sample fraction. However, other genes in the pathway overlapped with the study by Mihi et al., that showed the gene *ADAM17* to be involved in the gastric cell population response during infection [27]. The upregulated gene inactive rhomboid protein 1 (*RHBDF1*) (log2fc 1.92) is a regulator of *ADAM17* as well as involved in cell proliferation and inflammation. At 21 dpi there was evidence of host oxidative stress leading to an immune response based in antioxidant gene activity. The antioxidant activity (GO:0016209) (Table 1) observed at 21 dpi may be the product of host immune responses such as ROS or possibly through lipid peroxidation credited to the parasite’s acquisition of host resources. The antioxidant pathway was being upregulated at 21 dpi seemingly driven leukotriene C4 synthase (*LTC4S*) (log2fc = 1.96). The gene *LTC4S* is a cellular oxidant detoxicant, involved in lipid and glutathione metabolism, specifically glutathione peroxidase activity. Three other genes involved in glutathione peroxidase activity in the host also populated the pathway. These were glutathione peroxidase 1, 3, and 4 (*GPX1*) (log2fc = 1.44), (*GPX3*) (log2fc = 1.15), (*GPX4*) (log2fc = 1.02).

Upregulation of these genes and pathways may arise from the response to oxidative damage requiring the host to modulate glutathione, eventually leading to an anti-inflammatory response [23]. Also present at 21 dpi only, as evidence of a lack of infected cattle being able to maintain proper nutrient acquisition. The Reactome term metabolism of vitamins and cofactors (REAC:R-BTA-196854) appears with multiple upregulated genes that drive negative regulation of multiple functions. This may indicate losses in macro- and micronutrient absorption efficiency. Genes poly (ADP-ribose) polymerase family, member 10 (*PARP10*) (log2fc = 1.56) is a negative regulator of multiple functions, while two upregulated lipoprotein receptors LDL receptor related protein 1 and 10 (*LRP1*) (log2fc = 1.19), (*LRP10*) (log2fc = 1.12) also stood out. The genes *LRP1* and *LRP10* interact with glycosyltransferases shown to play roles in *Ostertagia* infections [24]. At 21 dpi there was evidence of issues in chemokine signaling, as most were downregulated. The genes C-X-C motif chemokine ligand 8 (*CXCL8*) (log2fc = −1.92), C-X-C motif chemokine ligand 2 (*CXCL2*) (log2fc = −1.93), C-X-C motif chemokine ligand 3 (*CXCL3*) (log2fc = −1.64), and atypical chemokine receptor 4 (*ACKR4*) (log2fc = −1.97). The gene *ACKR4* controls chemokine levels and promotes high-affinity binding leading to chemokine sequestration and degradation. Apoptotic clearance is also taking place at 21 dpi, this point the parasite is in its early adult stage and unique cell destruction pathways related to the mitochondria (regulation of mitochondrial outer membrane permeabilization involved in apoptotic signaling pathway GO:1901028) and intrinsic signaling pathways (negative regulation of intrinsic apoptotic signaling pathway GO:2001243) may take place. Despite the negative regulation, the host is still able to maintain efferocytosis (KEGG:04148), the clearance and elimination of apoptotic cells. However, efferocytosis can play a role in inflammatory responses as well as aid in clearance [30]. Within this pathway at 21 dpi is a different make-up of genes in action than at 14 dpi.

### 3.3. Gene Expression Analysis Results for 0 Dpi vs. 26 Dpi Timepoint Comparison

By 26 dpi, the parasite is in its adult stage. The number of DEGs is 810 with 55% upregulated. The big difference compared to the other timepoints is that the results start to show evidence of healing. The GO term wound healing (GO:0042060) (Table 1) shows up at 26 dpi. Fifteen of the sixteen genes that mapped to that term were upregulated, possibly indicating a drive towards healing in the host GI tract. Some of the genes driving this term included glycoprotein 1b platelet subunit beta (*GP1BB*) (log2fc = 2.87), which acts in humans to promote blood coagulation and platelet adhesion to exposed collagen [19,23,24]. The gene dysferlin (*DYSF*) (log2fc = 1.4) which plays a role in repair of both skeletal muscle resealing when membranes are disrupted by mechanical stress, which could result from motile adult stage parasites [19,24]. However, the gene fibroblast growth factor 2 (*FGF2*) (log2fc = −1.43); a heparin-binding integrin ligand plays a key part in the regulation of cell survival, division, differentiation, and migration. The gene also functions as a potent chemoattractant [19,23,24]. Its downregulation in this study could be the result of M2 macrophage polarization.

These genes also appear upregulated under the term functional abnormality of the GI tract (HP:0012719). This timepoint showed evidence of a tug of war between the positive regulation of defense response (GO:0031349) and negative regulation of the defense response (GO:0031348). While the terms overlapped by five genes (*DHX58*, *ARG1*, *NR1H3*, *TREX1*, and *TNFAIP3*), there were unique genes linked to positive regulation such as NF-kappa-B inhibitor zeta (*NFKBIZ*) (log2fc = −1.53) an inhibitor of NF-kappa-B transcription factor complexes. At 26 dpi the host still showed evidence of chemokines being downregulated (*CXCL8*, log2fc = −3.11) (*CXCL3*, log2fc = −3.01) (*CXCL2*, log2fc = −2.59). However, now it is expressed in conjunction with the gene immediate early response 3 *(IER3*) (log2fc = −1.16) that in human and mouse is a negative regulator of apoptosis, inflammation, glycolytic processes and is involved in the immune response to viral GI pathogens [31]. Observed at 26 dpi, the term malaria (KEGG:05144) links the host immune system to a different parasite infection but opens the door for possible overlap with future treatments. The genes under the term included a chemokine *CXCL8* (log2fc = −3.31) and *HBB* (log2fc = 4.54) and HBA (log2fc = 6.92). Another upregulated gene from the term was selectin P (*SELP*) (log2fc = 1.43) which functions to enable binding to the DAMPs heparin and sialic acid [24,32].

### 3.4. Gene Expression Analysis Results for Genes That Overlapped Across All Timepoints

**Figure 1:**
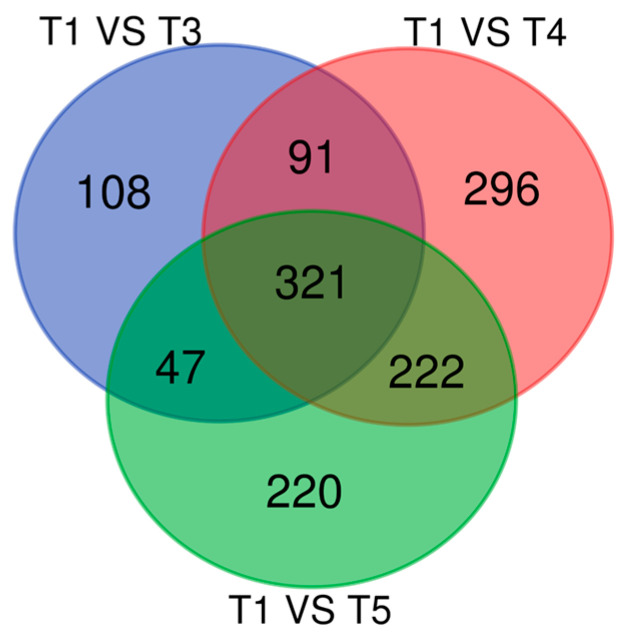
Venn diagram showing the number of shared genes between timepoints. T3 (14 dpi), T4 (21 dpi), and T5 (26 dpi). Overlapping genes (n = 321) were examined to mark the GO terms and pathways shared by all timepoints.

A Venn diagram analysis (Figure 1) was performed to identify genes that exhibited differential expression across the gene lists for 14 dpi, 21, dpi, and 26 dpi. A total of 321 (Supplementary Table S1) genes fell within this shared grouping. There were 112 genes being downregulated across the three timepoints leaving the majority of the list, 209 genes, upregulated. The timepoint with the highest fold changes and the most upregulated genes shared across all timepoints was 21 dpi (Supplementary Figure S1). This included many antiviral genes such as *RSAD2* and *ISG15*. The most upregulated genes in this list were the hemoglobin genes *HBA*, *HBB*, and *HBM* respectively. Most of the heavily downregulated genes only had Ensembl gene IDs and appeared as novel genes in cattle. Though they are novel genes some are recognized as long non-coding RNAs (LncRNAs) based on their gene type. One other downregulated gene of interest is phospholamban (*PLN*) which functions as a negative regulator in calcium transport.

All the shared DEGs were used to perform GO and pathway analysis to uncover genomic trends that lie beneath the entire course of the infection. When the GO terms and fold changes were explored further key immune pathways were revealed to be up regulated. This led to observations that the three timepoints (14 dpi, 21 dpi, 26 dpi) were upregulated for the GO term defense response to symbiont (GO:0140546). Additionally, the GO terms defense response to symbiont (GO:0140546), abnormality of the gastrointestinal tract (HP:0011024), and biological process involved in interspecies interaction between organisms (GO:0044419) resulted from the overlapping genes (n = 321) from all timepoints draw attention to the host immune system’s engagement with *O. ostertagi* at all stages and timepoints (Table 2).

**Table 1:** List of pathways and GO terms. Shows multiple terms that relate to possible host healing with overlap of terms and genes.

T1 vs. T3	G.O./PATHWAY ANALYSIS	ID	Genes
	Killing by host of symbiont cells	GO:0051873	ARG1, ELANE, ROMO1
	Apoptotic signaling pathway	GO:0097190	NUPR1, SNAI1, PHLDA3, BBC3, SHISA5, FADD, HRAS, GPX1, ZNF385A, LMNA, BAK1, CEBPB, CDKN1A, SIVA1, RBCK1, GRINA, CD74, PPM1F, PRELID1, ENO1, TGFB1, BAD, YJEFN3, PELI3, PHIP, ATM, BRCA2
	Abnormality of the gastrointestinal tract	HP:0011024	ELANE, AGRN, ELN, TUBB4A, ENSBTAG00000050645, HSPG2, HRAS, SREBF1, ALDH4A1, CYBA, SLC25A1, PLXND1, NECTIN1, VWF, IFT27, LMNA, PLOD1, PSAP, ECM1, MRPS34, TUBA1A, MIF, NOTCH3, ENSBTAG00000011704, CDKN1A, CORO1A, RBCK1, GRN, AP2S1, FLNA, TREX1, GLYCTK, PAX8, CHCHD10, ARPC1B, B9D2, TGFB1, GNB2, PHGDH, ENSBTAG00000027075, HSD3B7, REV3L, SCLT1, DYNC2H1, PHIP, ATM, ARL6, ENSBTAG00000068505, LIG4, CEP290, ATRX, BRCA2, VPS13A, TMTC3
	Epithelium migration	GO:0090132	SNAI1, HB-EGF, GPX1, PLXND1, ZNF580, RRAS, NR4A1, CORO1A, CORO1B, PPM1F, TGFB1, VASH1
	Efferocytosis	KEGG:04148	ARG1, DUSP2, C1QA, NR1H3, AXL, CEBPB, LRP1, DUSP4, MFGE8, TGFB1
	Biological process involved in interspecies interaction between organisms	GO:0044419	ISG15, SIGLEC1, RSAD2, ARG1, CMPK2, ENSBTAG00000008021, ELANE, ISG20, IRF7, ZDHHC1, DHX58, ENSBTAG00000053806, CFD, ENSBTAG00000046944, FADD, HRAS, ZFP36, CFP, EMILIN1, GPX1, SLC15A3, CYBA, C1QA, NR1H3, NR4A1, ENSBTAG00000032450, AXL, ADAM15, MIF, BAK1, GSN, CEBPB, CORO1A, RBCK1, GRN, TREX1, BATF3, ENSBTAG00000017645, CD74, ZBED1, MAPKAPK3, NECTIN2, ENO1, TGFB1, TOLLIP, FCN1, PC, ROMO1, VAMP8, EEA1, ENSBTAG00000068505, GZMA, STXBP4, NEDD4, ENSBTAG00000047632
	Epithelial cell migration	GO:0010631	SNAI1, HB-EGF, GPX1, PLXND1, ZNF580, RRAS, NR4A1, CORO1A, CORO1B, PPM1F, TGFB1, VASH1
	Defense response to symbiont	GO:0140546	ISG15, RSAD2, ARG1, ENSBTAG00000008021, ELANE, ISG20IRF7, ZDHHC1, DHX58, ENSBTAG00000053806, CFD, ENSBTAG00000046944, FADD, CFP, SLC15A3, CYBA, C1QA, NR1H3, ENSBTAG00000032450, ADAM15, MIF, GSN, CORO1A, GRN, TREX1, ENSBTAG00000017645, CD74, MAPKAPK3, NECTIN2, TOLLIP, FCN1, ROMO1, STXBP4, NEDD4, ENSBTAG00000047632
**T1 vs. T4**	Defense response to symbiont	GO:0140546	CYBA, CFD, MUL1, GRN, TOLLIP, CD74, TFEB, FADD, CORO1A, TREX1, CFP, MAPKAPK3, ADAM15, GSN, SLC15A3, ZYX, ROMO1, NLRX1, NR1H3, IFI35, CDC42EP4, FGR, MARCHF2, MIF, LAMP1, ENSBTAG00000046944, SENP7, PYCARD, NEDD4, MASP2, ENSBTAG00000017645, ISG15, IRF7, MOV10, NECTIN2, DHX58, ENSBTAG00000011961, UBA7, CXCL3, RAB20, FCN1, SRC, GRO1, TRIM59, RSAD2, C1QA, ISG20, MX2, ENSBTAG00000047632, S100A8, ZBP1, RAB7B, CXCL2, IFI6, ENSBTAG00000053806, CSF1, IFIT2, ARG1, OAS1Y, IFITM3
	Epithelial cell migration	GO:0010631	GPX1, CORO1B, RRAS, CORO1A, AKT3, TGFB1, PPM1F, HSPB1, HMOX1, ZNF580, VASH1, ACVRL1, PLXND1, SRC, JUP, S100A2, SNAI1
	Abnormality of the gastrointestinal tract	HP:0011024	HRAS, GNB2, CYBA, AP2S1, ARPC1B, ALDH4A1, SLC35C1, GRN, SREBF1, CRKLSLC25A1, RBCK1, CORO1A, TREX1, MRPS34, PSAP, IFT74, SPINT2, VWF, EDEM3, ECE1, FLNA, TTBK2, COMT, GLYCTK, PHIP, ABCA3, TGFB1, NCF1, TNFRSF1A, AHCY, SELENON, MYL9, B9D2, MIF, TYROBP, TMTC3, CSF1R, HMOX1, BRCA2PLOD1, NECTIN1, NOTCH3, REV3L, ATRX, SGO1, DYNC2H1, SPIB, SLC13A5, ENSBTAG00000007816, IL1RN, ECM1, GP1BB, ACVRL1, ELN, CENPF, SERPINE1, MASP2, CEP290, DYSF, PYGM, VPS13A, AGRN, ENSBTAG00000027075, ARL6, CHCHD10, PLXND1, LAMB2, LMNA, BOLA-DQB, HSPG2, ENPP1, SRC, ENSBTAG00000050645, EPCAM, IFT56, CDKN2A, TUBB4A
	Antioxidant activity	GO:0016209	GPX1, GPX4, HBB, HBA, GPX3, ALOX5AP, LTC4S, S100A8
	Biological process involved in interspecies interaction between organisms	GO:0044419	HRAS, CYBA, GPX1, CFD, MUL1, ZBED1, ENO1, VAMP8, GRN, TOLLIP, CD74, TFEB, FADD, VPS18, RBCK1CORO1A, TREX1, CFP, MAPKAPK3, ADAM15, BAK1, GSN, SLC15A3, ZYX, ROMO1, NLRX1, TGFB1, NR1H3, IFI35, CDC42EP4, IL17RC, FGR, TNFRSF1A, MARCHF2, HSPB1, MIF, LAMP1, CSF1R, ENSBTAG00000046944, EEA1, SENP7, PYCARD, CEBPB, NEDD4, CXCL8, CCDC186, MASP2, ENSBTAG00000017645, ISG15, IRF7, BATF3, SIGLEC1, MOV10, NECTIN2, PC, DHX58, ENSBTAG00000011961, UBA7, SMAD6, CXCL3, RAB20, FCN1, SRC, GRO1, EMILIN1, TRIM59, RSAD2, C1QA, ISG20, MX2, ENSBTAG00000047632, S100A8, ZBP1, RAB7B, CXCL2, IFI6, C5AR1, ENSBTAG00000053806, CSF1, CMPK2, IFIT2, ARG1, OAS1Y, IFITM3
	Metabolism of vitamins and cofactors	REAC:R-BTA-196854	LRP10, SLC19A1, TCN2, MOCS1, PARP10, PDXK, LRP1, AMN, AGRN, PC, ENPP1, SDC3, SDC4
**T1 vs. T5**	Wound healing	GO:0042060	HRAS, GPX1, VWF, HPS6, GP9, FLNA, CORO1B, MPIG6B, HMOX1, GP1BB, ENSBTAG00000047175, TUBB1, DYSF, SELP, SDC4, FGF2
	Functional abnormality of the gastrointestinal tract	HP:0012719	GNB2, HRAS, SLC35C1, ARPC1B, MRPS34, CORO1A, VWF, SLC25A1, ACTB, MIF, ALDH4A1, IFT74, RBCK1, TREX1, MYL9, GP9, FLNA, NFKBIA, TGFB1, TTBK2, PSAP, TNFAIP3, TMTC3, IL7R, ECE1, TUBB4A, HMOX1, SPIB, NOTCH3, ECM1, LMNA, CEP290, ATRXGP1BB, MASP2, NCF1, ELN, MVK, DTYMK, VPS13A, HSPG2, PHGDH, BRCA2, AGRN, CDKN2A, DYSF, NRCAM, PYGM
	Malaria	KEGG:05144	CXCL8, TGFB1, GYPC, HBA, LRP1, HBB, SELP
	Defense response to symbiont	GO:0140546	MUL1, FADD, TOLLIP, CORO1A, CYBA, EXOSC4, CXCL3, ROMO1, CFD, MIF, NFKBIZ, NR1H3, CD74, TREX1, CFP, GRO1, NFKBIA, MAPKAPK3, MSRB1, CDC42EP4, GSN, SLC15A3, CCL4
	Biological process involved in interspecies interaction between organisms	GO:0044419	HRAS, ENO1, BAK1, ZBED1, MUL1, CXCL8, FADD, TOLLIP, CORO1A, GPX1, CYBA, EXOSC4, CXCL3, ROMO1, CFD, MIF, NFKBIZ, RBCK1, NR1H3, THOC6, CD74, TREX1, CFP, GRO1, NFKBIA, TGFB1, MAPKAPK3, MSRB1, CDC42EP4, GSN, SLC15A3, CCL4, ENSBTAG00000032450, ENSBTAG00000011961, IFI35, MARCHF2, TNFAIP3, IL7R, HYAL2, ENSBTAG00000017645, IER3, IL17RC, CXCL2, MOV10, MASP2, IRF7, ISG15, ISG20, PYCARD, RAB7B, CDC42EP2, ABCA1, SIGLEC1, BATF, ENSBTAG00000008021, RSAD2, C1QA, NECTIN2, ENSBTAG00000053806, FCN1, RAB20, ENSBTAG00000046944, CMPK2, S100A8, UBA7, IFIT2, IFIT3, ARG1, IFITM3, MX2, ZBP1, IFI6, ZDHHC1, DHX58, LRRC19, LAG3, IL27

## 4. Discussion

Observations point to PBMCs having the ability to show a system-wide host response observable in blood across timepoints. Analysis of all the genes shared across timepoints (Figure 1) revealed the host immune response was mainly one of defense against the larval and adult stages of parasitic infection. Some of the more potent immune-related genes showed an increase in expression across the timepoints, while most others showed a peak in expression on day 21. This may be due to 21 dpi being the point in the parasite’s life cycle when the adult parasite exits the gastric glands and causes tissue damages. One gene that follows this trend of increased expression across the infection, DExH-box helicase 58 (*DHX58*), appears to work against the host as a negative regulator against the defense response and type I interferon production [19,23,24]. Although *DHX58* appears to favor the parasite, it may have still aided the host through processes like its involvement in the cytoplasmic pattern recognition receptor signaling pathway. Many type I interferons were expressed during the *O. ostertagi* infection, appearing to indicate that the parasite also stimulates upregulation of antiviral immune responses. The genes *ISG15*, *RSAD2*, *ISG20*, *IRF7* were all upregulated across timepoints and in multiple pathways. This could be part of the processes driving inflammation in cattle during parasitic infection. Although mostly attributed to antiviral functions, the DEG results indicate that they do act in anti-parasitic defense responses. This trend was shown to happen in sheep infected with *Teladorsagia circumcincta*, a nematode parasite closely related to *O. ostertagi*. Although many of the highlighted genes are best known for being anti-viral, Ahmed et al., 2015 showed that many of these genes are also differentially expressed in the ovine abomasa lymph node during GI parasite infections [33]. The genes in the ovine study fell into GO terms related to the immune and defense response and have overlap with what our current study observed in the host PBMCs. Both studies show that the genes *RSAD2* and *ISG20* have a role in the immune response to GI parasites.

Additionally, extracellular *ISG15* has also been indicated as being involved in the immune response to *Toxoplasma gondii* showing again that some viral related genes have the ability to assist in defense against GI nematode parasites [34]. This may signal the ability of the host to incorporate free-circulating *ISG15* and *ISG20* to recruit cytokines to inhibit parasite growth. The GO term biological process involved in interspecies interaction between organisms (GO:0044419) gave an example of the host interactome across timepoints based on gene interactions that fall within these several pathways. Upregulated genes (Table 1) related to antiviral functions showed up alongside a few genes that act as peroxidases that resolve ROS stress like *GPX1,* falling in line with antioxidant activity established at 21 dpi. Also unique to this GO term was the upregulated gene sialic acid binding Ig like lectin 1(*SIGLEC1*) which is activated in monocytic cells to interact with the gene mucin 1 (*MUC1*); a gene shown to respond during GI nematode infections [24,29]. Using the human phenotype database (HP) to examine the function and pathway terms related to the genes shared across all timepoints, led to the pathway term abnormality of the GI tract (HP:0011024). This term consisted of 30 shared genes, 25 of which were upregulated, indicating turmoil in the host GI tract. Expression tended to increase with each timepoint, however in some cases the expression at 21 dpi was the highest. This may indicate that the change from L5 to the adult stage and exiting of worms to the stomach lumen caused mass instability within the GI of the host. This transition may have activated DAMP signaling [7] through its ability to perform chondroitin sulfate and heparan sulfate proteoglycan binding in the host, leading to or possibly caused by genes such as *AGRN*, shown to be expressed as part of the plasma membrane during GI parasite infections in sheep [19,24,32,33]. Overall, the expression changes indicate systemic immune responses that affect the entire animal and can be detected in PBMCs during *Ostertagia* infection.

The host PBMCs show immune signaling that impacts the bovine GI system differently at each parasite life stage. Evidence emerged in the study that circulating PBMCs were picking up cues to what was happening in the host abomasum at each timepoint. At 14 dpi apoptotic clearance was occurring in the host with upregulation of the T-cell apoptotic process (GO:0070231) it is possible that there was a significant T-cell turnover as a defense response to the different life cycle stages, which may have been driven by the release secretory proteins from adult *O. ostertagi*. Increased apoptosis in the macrophages was shown to be affected by the parasite secretory molecules. However, the host may be increasing apoptosis due to neutrophil responses. This could be related to expression of a gene unique to 14 dpi, elastase neutrophil expressed (*ELANE*) (log2fc = 2.16), an inflammatory gene involved in neutrophil mediated killing. Overexpression of *ELANE* at 14 dpi may possibly be driving additional immune responses that led to an increase in apoptotic neutrophils in the host [35]. Additionally, 14 dpi apoptotic clearance could be linked to the upregulation of the GO terms epithelial cell proliferation (GO:0050673) and epithelial cell migration (GO:0010631). The latter containing the upregulated gene, *HB-EGF* (log2fc = 1.35), shown to play a role in *Ostertagia* infection of gastric epithelial cells (Table 2) [27,36]. This might indicate that PBMCs can inform researchers on immune responses to parasites residing in the GI system. It is also feasible that the resolution of apoptotic clearance by the efferocytosis pathway (KEGG:04148), present at 14 and 21 dpi, was also related to the immune reaction at the infection site. Multiple genes in the efferocytosis pathway have been shown to play a role in the response to GI nematodes. The genes *ARG1* and complement C1q A chain (*C1QA*) are upregulated within the pathway at both timepoints. The gene *ARG1* is possibly inhibiting the change over from innate to adaptive immunity due to its negative regulation of T-cell proliferation, type II interferon signaling, and T-helper 2 (Th2) cytokine production [23,24]. Perhaps the cattle differential expression at 14 dpi is set-up to disrupt the parasite life cycle by reduced expression of signaling and receptor activity against parasite attachment and feeding.

The apoptosis and efferocytosis at 14 and 21 dpi along with host ROS utilization to attack the parasite likely led to oxidative stress production in the host. Oxidative stress has been shown to be a significant component of parasitic infection as the host produces oxidants to counter the infection. The parasite, however, can cause added stress to the host as it usurps host resources leading to lipid peroxidation. Increases in oxidative stress can result in free radical production that eventually damages the host [26,37]. Although the parasite has transitioned from the L5 to the adult stages at 21 dpi, this imbalance in oxidative stress is observable in the blood. Oxidative stress led to the results exhibiting upregulation of the GO term antioxidant activity (GO:0016209) revealing that the host was actively dealing with system wide dysregulation. Genes such as *HBA* and *HBB* indicated the connection with the PMBCs while others, *GPX1* and arachidonate 5-lipoxygenase activating protein (*ALOX5AP*), were involved in cellular oxidant detoxification. The signal for this action may be through the DAMP, S100A8, being picked up in the PBMCs [19,24,32]. The genes observed within the antioxidant pathway overlapped with each other around molecular functions related to glutathione activity and lipid binding and metabolism. This could be a product of *O. ostertagi* infection within the abomasum of the cattle partially driven by epithelial turnover implied at 14 dpi.

Another pathway indicating the PBMC’s ability to carry host–parasite interaction signals originating in the GI was the GO term wound healing (GO:0042060). This term is not seen prior to 26 dpi and may be related to the type 2 immune response abrogating tissue damage and cell destruction [29,33,38] caused by the adult stage of *O. ostertagi*. A couple of genes from this upregulated pathway appear to be linked to the healing of the cattle GI. The gene milk fat globule EGF and factor V/VIII domain containing (*MFGE8*) (log2fc = 1.83) is associated with wound healing and an integral part of intestinal epithelial homeostasis where it spurs mucosal healing. The gene *GP1BB* (log2fc = 2.87) in humans is annotated to be involved in clotting and coagulation reflecting possible repair to the cattle GI system. Overall, these observations may indicate a secondary immune signaling mechanism against *Ostertagia* may occur in DAMPs along with previous studies showing immune activation in TLRs [5]. Though the observations in this study point to the utility of examining host PMBCs, future studies will need more animals and longer reads to pin down what is occurring in the host. Also, the genes active during our study occurred in animals naïve to parasite exposure. The expression might be different in animals that are pasture raised or previously exposed to parasites multiple times.

## 5. Conclusions

Overall, the transcriptomic changes observed in the PBMCs showed that cattle experiencing *O. ostertagi* infections carry clues that can be observed in their blood as to the severity and life stage of the parasitic infection. The host differential expression across timepoints indicated that the host is fighting against the parasite infection. However, subsequent change to adult parasites and the transition from the gastric glands to the abomasal lumen prompts a change in host responses towards wound healing. The results revealed that the host immune response and associated tissue damage gene expression corresponded with the different parasite life stages. The observation that the host upregulates many of the same genes with antiviral functions could mean the host is dual purposing some antiviral gene expression in control of parasite replication/growth. The upregulated anti-viral genes could be the result of host inflammatory response or possibly parasite caused opportunistic vir1al infection. However, additional studies are needed using more animals, increased timepoints that capture more of the host responses caused by L3 and L4 larvae, and any possible phenotypic data to help unravel the total ability of PBMCs against GI nematodes.

## Figures and Tables

**Table 2:** Terms based on pathway analysis of the genes shared by all 3 timepoints. Genes in **red** are shared by all 3 terms. Results show a common theme of the host actively dealing with the parasitic infection at earlier timepoints in the study prior to switching to tissue repair at 26 dpi (T5).

**Biological process involved in interspecies interaction between organisms (GO:0044419)**				
**GENE ID**	**GENE NAME**	**T3**	**T4**	**T5**
ENSBTAG00000014707	ISG15	3.69	4.71	4.33
ENSBTAG00000013167	SIGLEC1	3.12	3.36	3.01
ENSBTAG00000016061	RSAD2	2.52	3.04	3.16
ENSBTAG00000019979	CMPK2	2.36	2.64	2.76
ENSBTAG00000014762	ISG20	2.08	2.44	2.75
ENSBTAG00000012403	ARG1	2.37	2.42	2.34
ENSBTAG00000047680	IRF7	1.98	2.35	2.23
ENSBTAG00000046944	ENSBTAG00000046944	1.31	2.10	1.33
ENSBTAG00000046580	DHX58	1.55	2.06	1.62
ENSBTAG00000054195	GPX1	1.23	1.44	1.24
ENSBTAG00000048122	CFD	1.40	1.43	1.15
ENSBTAG00000015815	CFP	1.25	1.41	1.29
ENSBTAG00000046644	HRAS	1.29	1.41	1.37
ENSBTAG00000053806	ENSBTAG00000053806	1.44	1.40	1.54
ENSBTAG00000061619	CYBA	1.23	1.38	1.14
ENSBTAG00000018274	FADD	1.29	1.35	1.40
ENSBTAG00000015318	NECTIN2	1.04	1.34	1.18
ENSBTAG00000008406	TREX1	1.06	1.32	1.19
ENSBTAG00000018366	SLC15A3	1.23	1.30	1.17
ENSBTAG00000017002	RBCK1	1.09	1.27	1.15
ENSBTAG00000016532	MAPKAPK3	1.04	1.22	1.07
ENSBTAG00000015228	CD74	1.05	1.21	1.00
ENSBTAG00000061194	ZBED1	1.05	1.20	1.17
ENSBTAG00000019915	GSN	1.10	1.20	1.08
ENSBTAG00000010681	NR1H3	1.16	1.16	1.24
ENSBTAG00000013411	ENO1	1.03	1.12	1.19
ENSBTAG00000008631	CORO1A	1.09	1.10	1.16
ENSBTAG00000000428	BAK1	1.11	1.10	1.37
ENSBTAG00000007153	C1QA	1.22	1.07	1.10
ENSBTAG00000008237	TOLLIP	1.02	1.06	1.02
ENSBTAG00000017645	ENSBTAG00000017645	1.06	1.04	1.14
ENSBTAG00000048155	FCN1	1.02	1.03	1.01
ENSBTAG00000020457	TGFB1	1.03	1.03	1.04
ENSBTAG00000027361	ROMO1	1.01	1.02	1.14
ENSBTAG00000007375	MIF	1.12	1.00	1.20
**Defense response to symbiont (GO:0140546)**				
**GENE ID**	**GENE NAME**	**T3**	**T4**	**T5**
ENSBTAG00000014707	ISG15	3.69	4.71	4.33
ENSBTAG00000016061	RSAD2	2.52	3.04	3.16
ENSBTAG00000014762	ISG20	2.08	2.44	2.75
ENSBTAG00000012403	ARG1	2.37	2.42	2.34
ENSBTAG00000047680	IRF7	1.98	2.35	2.23
ENSBTAG00000046944	ENSBTAG00000046944	1.31	2.10	1.33
ENSBTAG00000046580	DHX58	1.55	2.06	1.62
ENSBTAG00000048122	CFD	1.40	1.43	1.15
ENSBTAG00000015815	CFP	1.25	1.41	1.29
ENSBTAG00000053806	ENSBTAG00000053806	1.44	1.40	1.54
ENSBTAG00000061619	CYBA	1.23	1.38	1.14
ENSBTAG00000018274	FADD	1.29	1.35	1.40
ENSBTAG00000015318	NECTIN2	1.04	1.34	1.18
ENSBTAG00000008406	TREX1	1.06	1.32	1.19
ENSBTAG00000018366	SLC15A3	1.23	1.30	1.17
ENSBTAG00000016532	MAPKAPK3	1.04	1.22	1.07
ENSBTAG00000015228	CD74	1.05	1.21	1.00
ENSBTAG00000019915	GSN	1.10	1.20	1.08
ENSBTAG00000010681	NR1H3	1.16	1.16	1.24
ENSBTAG00000008631	CORO1A	1.09	1.10	1.16
ENSBTAG00000007153	C1QA	1.22	1.07	1.10
ENSBTAG00000008237	TOLLIP	1.02	1.06	1.02
ENSBTAG00000017645	ENSBTAG00000017645	1.06	1.04	1.14
ENSBTAG00000048155	FCN1	1.02	1.03	1.01
ENSBTAG00000027361	ROMO1	1.01	1.02	1.14
ENSBTAG00000007375	MIF	1.12	1.00	1.20
**Abnormality of the gastrointestinal tract (HP:0011024)**				
**GENE ID**	**GENE NAME**	**T3**	**T4**	**T5**
ENSBTAG00000013191	AGRN	1.94	2.52	2.17
ENSBTAG00000012265	VWF	1.18	2.32	2.55
ENSBTAG00000019517	ELN	1.44	1.57	1.45
ENSBTAG00000017574	LMNA	1.16	1.49	1.73
ENSBTAG00000030335	ALDH4A1	1.26	1.46	1.15
ENSBTAG00000046644	HRAS	1.29	1.41	1.37
ENSBTAG00000001814	PLXND1	1.20	1.40	1.00
ENSBTAG00000043971	NOTCH3	1.12	1.38	1.31
ENSBTAG00000017122	HSPG2	1.31	1.38	1.45
ENSBTAG00000061619	CYBA	1.23	1.38	1.14
ENSBTAG00000008406	TREX1	1.06	1.32	1.19
ENSBTAG00000017002	RBCK1	1.09	1.27	1.15
ENSBTAG00000021499	PSAP	1.13	1.23	1.10
ENSBTAG00000011190	FLNA	1.07	1.19	1.15
ENSBTAG00000010584	AP2S1	1.09	1.17	1.10
ENSBTAG00000008528	SLC25A1	1.21	1.17	1.11
ENSBTAG00000003806	ECM1	1.13	1.13	1.15
ENSBTAG00000026415	MRPS34	1.12	1.10	1.27
ENSBTAG00000008631	CORO1A	1.09	1.10	1.16
ENSBTAG00000023452	B9D2	1.04	1.08	1.14
ENSBTAG00000046248	ARPC1B	1.04	1.07	1.01
ENSBTAG00000021013	TUBB4A	1.40	1.07	1.73
ENSBTAG00000020457	TGFB1	1.03	1.03	1.04
ENSBTAG00000006495	GNB2	1.03	1.02	1.07
ENSBTAG00000007375	MIF	1.12	1.00	1.20
ENSBTAG00000018745	CEP290	−1.06	−1.12	−1.17
ENSBTAG00000017734	VPS13A	−1.24	−1.14	−1.05
ENSBTAG00000038434	ATRX	−1.13	−1.15	−1.03
ENSBTAG00000009023	TMTC3	−1.28	−1.39	−1.38
ENSBTAG00000000988	BRCA2	−1.13	−1.41	−1.02

## Data Availability

Availability in SRA PRJNA1221871.

## Supplementary Materials

The following supporting information can be downloaded at: https://www.mdpi.com/article/10.3390/biology14081034/s1, Supplementary Table S1. Gene list of overlapping genes from Venn diagram. Supplementary Figure S1. Heatmap of overlapping genes from Venn diagram.

## Abbreviations

The following abbreviations are used in this manuscript:

DPI	Days post infection
PBMC	Peripheral Blood Mononuclear Cell
