# Supplementary figures and images for: Transcriptomic Analysis of Peripheral Blood Mononuclear Cells During Ostertagia ostertagi Infection in Cattle Highlights a Generalized Host Immune Reaction
Source: Biology (Basel). 2025 Aug 12;14(8):1034. doi: 10.3390/biology14081034 (PMC12384011; doi:10.3390/biology14081034)

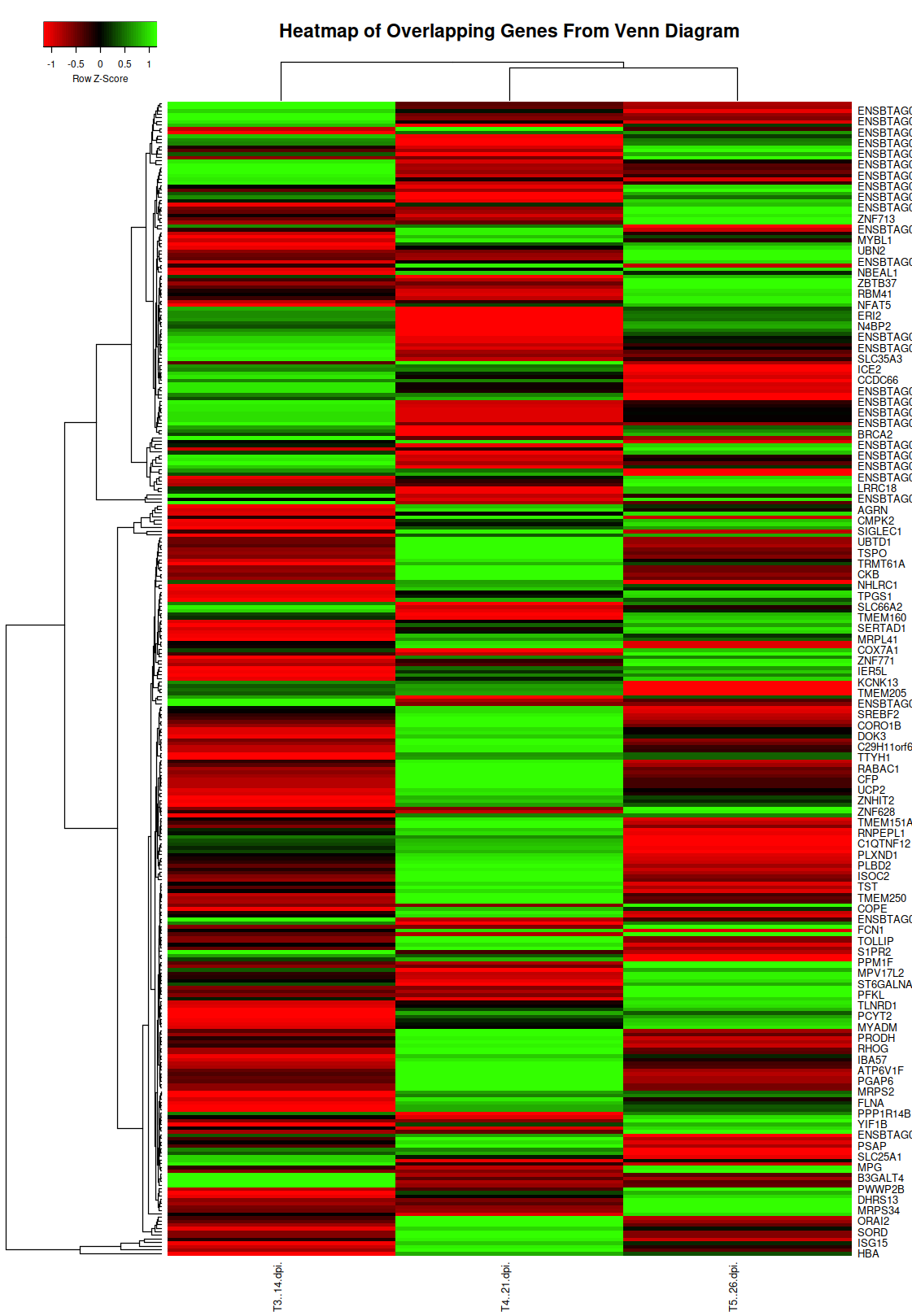

Supplement: Supplementary file 1 [file biology-14-01034-s001.zip › biology-3687309-Figure S1.png]
